# The association of mindfulness with stress self-management among university teachers: the mediating roles of resilience and cognitive reappraisal

**DOI:** 10.3389/fpsyg.2025.1679459

**Published:** 2025-11-07

**Authors:** Shuting Liao, Anbang Hu

**Affiliations:** 1 Hunan Industry Polytechnic, Changsha, China; 2 International College, National Institute of Development Administration, Bangkok, Thailand; 3 School of Public Administration, Hunan Normal University, Changsha, China

**Keywords:** mindfulness, resilience, cognitive reappraisal, stress self-management, University teachers

## Abstract

**Introduction:**

This study addresses a critical gap in understanding the psychological mechanisms underlying stress self-management among university teachers. Specifically, it develops and tests a Conservation of Resources (COR)-based dual-mediator model, examining how mindfulness contributes to stress self-management through two sequentially linked psychological resources: resilience and cognitive reappraisal. By focusing on this sequential pathway, the study provides novel insights into the dynamic resource accumulation process that supports adaptive stress regulation in higher education contexts.

**Methods:**

This study targets university teachers in Hunan Province. Using snowball and purposive sampling, participants were asked to forward the questionnaire to colleagues. The survey was conducted in June 2025, collecting 287 valid responses via Wenjuanxing. Data were analyzed using structural equation modeling (SEM) in AMOS 26.0 to examine the relationships among mindfulness, resilience, cognitive reappraisal, and stress self-management.

**Results:**

The analysis revealed significant associations among the study variables. Mindfulness was positively associated with both resilience (*β* = 0.469, *p* < 0.001) and cognitive reappraisal (*β* = 0.317, *p* < 0.001). Resilience was positively related to cognitive reappraisal (*β* = 0.561, *p* < 0.001) and stress self-management (*β* = 0.345, *p* < 0.001). Cognitive reappraisal was also positively associated with stress self-management (*β* = 0.366, *p* < 0.001). Moreover, resilience and cognitive reappraisal jointly mediated the relationship between mindfulness and stress self-management (indirect effect = 0.374, CI [0.270, 0.475], *p* < 0.001). R^2^ for Stress Self-Management is 0.43, indicating that mindfulness, resilience, and cognitive reappraisal together explain 43% of its variance.

**Discussion:**

These findings suggest that mindfulness is linked to higher resilience and greater use of adaptive cognitive strategies, which are in turn associated with better stress self-management. The study contributes to the application of COR theory in educational settings by identifying a sequential resource-activation pathway. Practically, the results indicate that mindfulness-based activities, combined with resilience training and emotion-regulation routines, may support psychological well-being and sustainable performance among university faculty. In sum, mindfulness is associated with better stress self-management largely through higher resilience and greater use of cognitive reappraisal.

## 1 Introduction

In recent years, university teachers have increasingly been recognized as a high-risk occupational group for stress and psychological distress. Although academic work is often perceived as intellectually fulfilling and flexible, the reality is that university faculty now face heavy workloads, extended working hours, and escalating performance pressures. These demands have led to alarming levels of burnout, anxiety, and depression among educators worldwide (Agyapong et al., 2022; Kush et al., 2022; Carvajal and Guedea, 2021). In China, these problems are particularly acute due to structural transformations in higher education. Intensified “publish or perish” and tenure-track systems, along with the rapid expansion of universities and the influx of young PhD graduates, have amplified intra-faculty competition and heightened job insecurity (Zhao and Ding, 2019; Li, 2013; Cao et al., 2023). Furthermore, administrative overload and frequent policy reforms add to teachers’ emotional exhaustion (Carroll et al., 2022). Collectively, these pressures make it increasingly difficult for Chinese university faculty to sustain psychological balance and long-term professional engagement.

However, under the increasingly performance-oriented environment of higher education, how university teachers cope with work-related stress at the internal psychological level has long been overlooked. In recent years, growing attention has been paid to how individual psychological resources enable teachers to regulate stress effectively (Kaya, 2024; Albright et al., 2024). Among these resources, mindfulness—a state of present-moment awareness and nonjudgmental acceptance—has been widely recognized as a key factor promoting mental health and emotional regulation (Almanasef and Almaghaslah, 2024). Existing research in China has shown that mindfulness enhances mental well-being among university students by fostering self-compassion and reducing rumination (Wu et al., 2025). However, compared with student populations, university teachers experience more complex and persistent forms of occupational stress (Meng and Wang, 2018; Ahmad et al., 2024), such as research evaluation (Gu and Levin, 2021), promotion pressure (Xu et al., 2023), and performance-based assessment (Pei et al., 2024), yet studies focusing on this specific professional group remain limited. Therefore, examining how mindfulness contributes to stress self-regulation among university faculty holds significant theoretical and practical value.

Most existing studies on teacher mental health have focused on primary and secondary school teachers or other high-stress professions such as healthcare workers (Albright et al., 2024; Miller et al., 2019; Prentice et al., 2024). Research on university teachers, by contrast, has largely remained at the descriptive level, documenting high levels of stress, anxiety, and burnout (Kallitsoglou and Mahmud, 2023; Vela et al., 2024; West and Shirley, 2024), while paying limited attention to the underlying psychological adaptation mechanisms. Moreover, many prior studies have examined psychological constructs in isolation, neglecting the dynamic interplay among multiple internal resources. To date, few empirical studies have integrated mindfulness, resilience, and emotion regulation strategies within a unified theoretical framework to systematically explain how university teachers mobilize internal psychological resources to achieve effective stress self-management.

Grounded in the Conservation of Resources (COR) theory (Hobfoll et al., 2018; Halbesleben et al., 2014), this study posits that psychological resources are central to coping with work-related stress. Mindfulness, as a foundational psychological resource, enables individuals to maintain awareness and acceptance in stressful contexts, thereby facilitating the generation and mobilization of other adaptive resources (Lucas-Thompson et al., 2024). Within this framework, resilience and cognitive reappraisal are conceptualized as key mediating mechanisms linking mindfulness to stress self-management. Resilience reflects an individual’s ability to recover and grow from adversity, while cognitive reappraisal represents a strategy for regulating emotions by reinterpreting stressful events. Both constructs align with the COR theory’s principle of “resource accumulation and mutual reinforcement.” Mindfulness activates individuals’ internal resources, enhances their capacity to cope with stress, and promotes adaptive cognitive and emotional regulation, ultimately leading to more effective stress management (Atta et al., 2024). Specifically, resilience serves as a crucial buffering resource that helps teachers recover from high-pressure situations and provides psychological support for emotional regulation (Almanasef and Almaghaslah, 2024). Cognitive reappraisal, in turn, represents a constructive emotion regulation strategy that reduces negative emotional experiences and strengthens psychological flexibility through the positive reinterpretation of events (Eisma et al., 2023). Based on these theoretical foundations, this study develops and tests an integrated model to examine how mindfulness influences university teachers’ stress self-management through the sequential mediating effects of resilience and cognitive reappraisal, thereby revealing the dynamic process of psychological resource accumulation.

The study makes three primary contributions. First, at the theoretical level, it empirically validates a “resource-building chain” pathway in which mindfulness enhances stress self-management through resilience and cognitive reappraisal, extending the explanatory power of COR theory in the fields of education and mental health. Second, at the contextual level, it expands the empirical investigation of mindfulness mechanisms from student populations to university faculty, enriching the understanding of how mindfulness, resilience, and emotion regulation interact within the Chinese cultural context. Third, at the practical level, the study identifies the key mechanisms underlying teachers’ psychological resource development, offering theoretical and actionable insights for promoting mental health, stress management, and emotion regulation training among university educators.

The following sections are structured as follows: Section 2 reviews relevant literature and presents the theoretical foundation and hypotheses. Section 3 details the research methodology. Section 4 reports the results of data analysis. Section 5 discusses the findings in relation to existing research, theoretical implications, and practical recommendations. Finally, Section 6 concludes the study with a summary and suggestions for future research.

## 2 Literature review and hypothesis development

### 2.1 COR theory

The COR theory, originally proposed by Hobfoll in 1989, has become a widely accepted theoretical framework for understanding the dynamics of stress and psychological adaptation (Hobfoll et al., 2018). This theory posits that individuals are inherently motivated to acquire, preserve, and protect resources that they consider valuable (Halbesleben et al., 2014). These resources include material assets such as tools and housing, personal characteristics such as self-efficacy and resilience, conditional factors such as stable employment and supportive social relationships, and energy-related resources such as time and knowledge (Potipiroon and Junthong, 2024; Li et al., 2024). Stress arises when individuals encounter the threat of resource loss, experience actual resource depletion, or fail to obtain expected returns after investing resources (Ng and Yeung, 2024). COR theory emphasizes that the psychological impact of resource loss tends to outweigh the benefits of resource gain, and individuals with fewer resources are more likely to experience a downward spiral of continuing resource loss (Dong et al., 2024; Qian et al., 2024).

In addition to this, COR theory introduces the notion of resource gain spirals. Individuals who possess greater initial resources are more capable of acquiring new resources, thereby improving their ability to cope with stress and maintain psychological well-being. This cumulative process suggests that resource-rich individuals tend to build on their strengths and adapt more effectively in high-stress environments (Liao et al., 2025; Schiff et al., 2025). In the field of occupational stress research, COR theory has been widely applied to examine how individuals respond to the pressures of work, particularly in high-demand sectors such as healthcare, education, and emergency services. Despite its widespread use, the application of COR theory to the university teaching profession has been relatively limited. Given the chronic, multi-faceted, and often ambiguous stressors faced by university faculty, including research expectations, heavy teaching loads, administrative responsibilities, and performance evaluations (Carvajal and Guedea, 2021).

The present study adopts the COR theory as its conceptual foundation to examine how university teachers draw upon internal psychological resources to manage stress. In this context, mindfulness is conceptualized as a foundational resource that enhances present-moment awareness and reduces automatic emotional reactivity (Cawley and Tejeiro, 2024). Resilience is viewed as a secondary resource that reflects the capacity to recover from setbacks and adapt over time (Kaya, 2024). Cognitive reappraisal, a widely studied emotion regulation strategy, is regarded as a strategic resource that enables individuals to reframe stress-inducing events in less threatening terms, thereby conserving psychological energy (Shum et al., 2025). These three psychological resources are interrelated and may jointly form an adaptive system that enhances stress self-management among university faculty. By situating these variables within the COR theoretical framework, this study aims to illuminate the psychological gain processes through which mindfulness fosters resilience and cognitive flexibility, ultimately supporting better stress regulation and emotional balance in academic settings.

### 2.2 Mindfulness, resilience, and cognitive reappraisal

Mindfulness, conceptualized as a present-focused, non-judgmental awareness of one’s experiences, has increasingly been recognized as a valuable psychological resource for stress reduction and mental well-being (Ueno and Amemiya, 2024). Empirical evidence indicates that mindfulness is associated with lower levels of anxiety and depression, and with enhanced emotional regulation and adaptability in stressful contexts (Almanasef and Almaghaslah, 2024; Berdida et al., 2023). From the perspective of COR theory, mindfulness can be regarded as a foundational individual resource that promotes the accumulation of further psychological resources by enhancing cognitive awareness and self-regulation.

Resilience refers to the psychological capacity to recover, adapt, and even grow in the face of adversity, stress, or traumatic events (Kaya and Odaci, 2024). Studies have shown that mindfulness training not only strengthens emotional regulation but also fosters greater cognitive flexibility when encountering stress, thereby facilitating the development of resilience (Le et al., 2024). A mindful state reduces overreaction to negative stimuli and enhances one’s capacity to accept and adapt to challenging circumstances. Therefore, individuals with higher levels of mindfulness are more likely to exhibit stronger resilience. Based on these theoretical and empirical foundations, the following hypothesis is proposed:

*Hypothesis* 1 (H1): Mindfulness is positively associated with resilience.

In addition, mindfulness has been identified as a significant antecedent of emotion regulation (Brockman et al., 2017; Hayes-Skelton and Graham, 2013). According to Pang et al. (2025) process model of emotion regulation, cognitive reappraisal is a form of antecedent-focused regulation, whereby individuals alter their emotional responses by changing how they interpret stress-inducing events. Mindfulness enhances both self-awareness and cognitive flexibility, enabling individuals to recognize and interrupt automatic thought patterns. As a result, they are more capable of cognitively reframing stressful situations in a constructive way, thereby mitigating negative emotional responses (Atta et al., 2024; Eisma et al., 2023). Research has confirmed that individuals with higher levels of mindfulness are more likely to employ cognitive reappraisal and are less likely to rely on maladaptive strategies such as suppression or avoidance (Giancola et al., 2024). Accordingly, the following hypothesis is proposed:

*Hypothesis* 2 (H2): Mindfulness is positively associated with cognitive reappraisal.

Moreover, individuals with higher levels of resilience typically exhibit more effective emotion regulation strategies, including a greater tendency to engage in cognitive reappraisal (Dolcos et al., 2021; Zhou and Li, 2022). Resilience is not only reflected in emotional stability and effective coping but also in the individual’s capacity to reframe and reinterpret adverse events in a more positive light (Han et al., 2023). Specifically, resilient individuals tend to maintain an optimistic perspective when facing emotional challenges and actively reconstruct the meaning of negative experiences to reduce psychological harm (Yang et al., 2024; Zhang et al., 2023). This process aligns closely with the cognitive reappraisal mechanism. Thus, the following hypothesis is proposed:

*Hypothesis* 3 (H3): Resilience is positively associated with cognitive reappraisal.

### 2.3 Resilience, cognitive reappraisal and stress self-management

Stress self-management refers to an individual’s active use of cognitive, behavioral, and emotional regulation strategies to cope with stressors encountered in daily life or work, aiming to mitigate their negative impact and maintain functional well-being (Wang and Tang, 2024). In the context of higher education, university faculty members are often exposed to persistent and multifaceted stressors, including teaching responsibilities, research productivity, promotion pressures, and administrative tasks (Carvajal and Guedea, 2021). Accordingly, the ability to effectively self-manage stress has become a critical psychological asset for maintaining professional functioning and occupational well-being.

Resilience, conceptualized as a form of psychological elasticity, plays a vital protective role in stress self-management (Bai et al., 2024). According to the COR theory, resilience constitutes a key internal resource that enables individuals to preserve or restore psychological balance when faced with threats of resource loss (Li and Yang, 2016). Empirical studies have shown that individuals with high resilience tend to exhibit greater adaptability, emotional stability, and constructive coping responses in stressful situations (Puertas-Gonzalez et al., 2023; Urban et al., 2022). Within the educational context, resilience helps educators cope with occupational demands, enhances their emotional regulation and interpersonal communication, and contributes indirectly to teaching effectiveness and professional satisfaction. Thus, the following hypothesis is proposed:

*Hypothesis* 4 (H4): Resilience is positively associated with stress self-management.

Cognitive reappraisal, as a constructive emotion regulation strategy, is also widely recognized as an effective mechanism for managing stress (Hale et al., 2024). It involves the process of reinterpreting or reframing the meaning of stressful events in order to reduce their emotional impact (Burnham and Kocovski, 2024). Research has consistently demonstrated that individuals who frequently utilize cognitive reappraisal tend to maintain a more positive emotional state under stress, experience lower levels of negative affect, and adopt more adaptive behavioral responses (Wolfe et al., 2023). For university teachers, cognitive reappraisal can facilitate psychological flexibility when facing student-related issues, research setbacks, or performance evaluations, thereby mitigating emotional exhaustion and enhancing professional efficacy. Thus, the following hypothesis is proposed:

*Hypothesis* 5 (H5): Cognitive reappraisal is positively associated with stress self-management.

### 2.4 Mediation effects

In recent years, mindfulness has increasingly been recognized as a key psychological resource that enhances individuals’ adaptive functioning and stress regulation (Oman, 2025). However, the underlying mechanisms through which mindfulness contributes to these outcomes require further investigation. According to the COR theory, mindfulness, as a core individual resource, can stimulate the accumulation of additional psychological resources, thereby promoting more effective coping outcomes (Zhang et al., 2024). Previous research suggests that mindfulness not only has a direct effect on reducing perceived stress, but may also indirectly influence stress responses through the enhancement of internal regulatory capacities (Kil and Grusec, 2020; Lucas-Thompson et al., 2024). Building upon this theoretical foundation, the present study proposes that resilience and cognitive reappraisal may function as mediators in the relationship between mindfulness and stress self-management, forming a dual mediation pathway model with greater explanatory power.

First, mindfulness can improve individuals’ awareness and emotional regulation in stressful situations, which in turn supports the development of resilience. Mindfulness practices help individuals remain calm in the face of adversity, reduce emotional volatility, and foster a more constructive attitude toward challenges (Minh Anh Quang et al., 2022). These outcomes form the psychological basis of resilience (Almanasef and Almaghaslah, 2024). Furthermore, individuals with high levels of resilience often display stronger emotional coping and problem-solving abilities, which are essential for maintaining effective stress self-management (Fenzel and Richardson, 2022; Kaya, 2024).

Second, mindfulness can significantly enhance cognitive flexibility, thereby promoting the use of adaptive emotion regulation strategies such as cognitive reappraisal (Martinez and Dong, 2020; Sinnott et al., 2020). Cognitive reappraisal helps individuals reduce the emotional impact of stressful events by encouraging reinterpretation of the situation in a more constructive light (Eisma et al., 2023). Through this mechanism, mindfulness indirectly facilitates improved stress management by increasing the likelihood that individuals engage in cognitive reappraisal.

**Figure 1:**
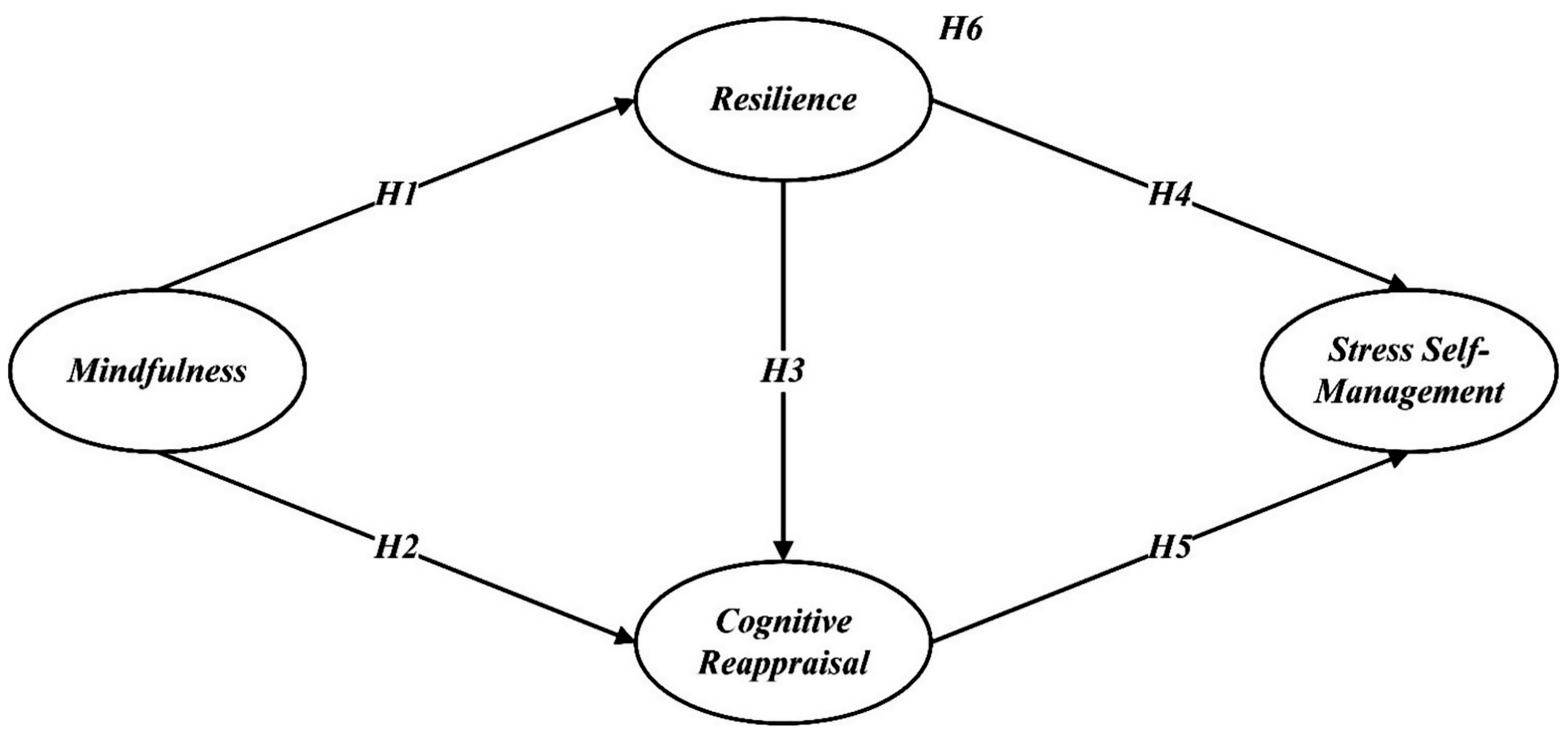
The hypothesized model.

Moreover, prior research has indicated that resilience and cognitive reappraisal are also positively related. Individuals with greater resilience are more likely to adopt cognitive reappraisal as a preferred strategy for handling negative emotions (Zhang et al., 2023). Based on this, it is plausible that mindfulness contributes to stress self-management through a multilayered resource activation pathway, mindfulness strengthens resilience, which in turn promotes cognitive reappraisal, ultimately enhancing stress coping capabilities. This type of sequential mediation pathway has not been extensively examined, particularly in the context of university faculty (Figure 1). Accordingly, the following hypothesis is proposed:

*Hypothesis* 6 (H6): Resilience and cognitive reappraisal mediate the relationship between mindfulness and stress self-management.

## 3 Methods

### 3.1 Participants and procedure

We surveyed university teachers in Hunan Province using snowball and purposive sampling. Department administrators at several universities were contacted to explain the study aims (mindfulness, resilience, cognitive reappraisal, and stress self-management), and recipients were invited to forward the link to colleagues. Participating institutions were primarily research-intensive public universities and key provincial institutions with strong publication and grant expectations, where faculty commonly face high research pressure. The online questionnaire (Wenjuanxing) was administered in June 2025. Inclusion criteria were: currently teaching at a Hunan university during data collection (tenured/tenure-track, contract, or adjunct/part-time), age ≥ 18, and ≥ 6 months of university-level teaching experience. Exclusion criteria were non-teaching staff or those with no teaching load in the current term, as well as data-quality failures (duplicate submissions via IP/device checks, failure on an attention-check item, straight-lining, excessive missingness) or incomplete questionnaires. In total, 302 questionnaires were returned; after quality screening, 287 valid cases remained (valid/returned completion = 287/302 = 94.7%). Because snowball sampling does not yield a well-defined population denominator, a conventional response rate is not estimable; we therefore report the completion ratio among returned questionnaires. All responses were anonymous and voluntary.

Table 1 presents the demographic profile of the university teacher sample in this study. The gender distribution is nearly balanced, with females slightly outnumbering males (53.0% vs. 47.0%). This slight difference might reflect the higher proportion of female employees in the education sector. Most participants are aged 35–45 years (45.6%), which suggests a stable and experienced group of educators, while the younger (under 35) and older (over 45) groups make up smaller proportions, possibly due to career stages and retirement patterns. In terms of marital status, the majority are married (60.6%), indicating the potential influence of family stability on career longevity in academia, while the proportion of singles (27.9%) reflects younger professionals or those without family obligations. Regarding teaching experience, the largest groups have 6–15 years (39.0%) or 0–5 years (27.2%), suggesting a balanced mix of experienced teachers and newcomers, which may offer a diverse range of perspectives in the study. Employment type shows that 56.8% are on contract, while 38.7% have permanent positions, possibly reflecting the growing use of contract-based staff in academia. Over half of the teachers work 40–50 h per week (54.0%), which may highlight the demanding nature of teaching and administrative duties in universities.

**Table 1:** Overview of participants.

Profiles	Frequency	Proportion
Gender		
Male	135	47.0%
Female	152	53.0%
Age		
<35	85	29.6%
35–45	131	45.6%
>45	71	24.7%
Marital status		
Single	80	27.9%
Married	174	60.6%
Divorced/Widowed	33	11.5%
Years of teaching experience		
0–5	78	27.2%
6–15	112	39.0%
16–20	45	15.7%
>20	52	18.1%
Employment type		
Tenured	111	38.7%
Contractual	163	56.8%
Adjunct/Part-time	13	4.5%
Average weekly working hours		
Less than 40 h	31	10.8%
40–50 h	155	54.0%
51–60 h	73	25.4%
Over 60 h	28	9.8%

### 3.2 Measures

Mindfulness was measured using the five-item Mindful Attention Awareness Scale (MAAS-5) developed by Van Dam et al. (2010), which includes items such as “I rush through activities without being really attentive to them.” Resilience was assessed with the six-item Brief Resilience Scale (BRS) by Smith et al. (2008), featuring statements like “I tend to bounce back quickly after hard times.” Cognitive reappraisal was evaluated using six items from the short form of the Emotion Regulation Questionnaire (ERQ-S) developed by Preece et al. (2023), for example, “I control my emotions by changing the way I think about the situation I’m in.” Stress self-management was measured using the six-item scale developed by Pinar et al. (2009), including items such as “Use stress control methods.” All instruments maintained their original structure and employed a five-point Likert scale ranging from 1 (“strongly disagree”) to 5 (“strongly agree”).

All instruments were administered in Chinese following a rigorous translation–adaptation procedure: items were translated by a certified professional translator, independently back-translated by two bilingual researchers, and reconciled by an expert panel to ensure conceptual equivalence and cultural appropriateness. We then conducted a pilot with 40 university teachers; all scales achieved Cronbach’s *α* > 0.80, after which the full survey was launched.

### 3.3 Data analysis

The hypothesized model was analyzed using structural equation modeling (SEM) in AMOS 26.0 with maximum likelihood (ML) estimation; standardized coefficients are reported. All items were 5-point Likert and were treated as approximately continuous after distributional checks. AMOS normality assessment showed acceptable univariate normality (max |skew| = 1.240; max |kurtosis| = 1.829). Missing data were not applicable because the Wenjuanxing platform enforced complete responses. To obtain robust inferences and test indirect effects, we used bias-corrected bootstrapping (5,000 resamples, two-tailed 95% CIs).

### 3.4 Common-method variance

Procedural remedies to curb common-method variance (CMV) were implemented ex-ante. The survey assured anonymity and confidentiality, emphasized that there were no right or wrong answers to minimize evaluation apprehension. Items were randomized and constructs were presented in separate blocks to achieve psychological separation between predictors (mindfulness, resilience) and outcomes (cognitive reappraisal, stress self-management). Reverse-worded items embedded in the scales further reduced acquiescence bias. We screened and removed inattentive or duplicate responses based on pre-specified criteria (incomplete patterns, duplicate IDs, abnormal completion time).

To statistically assess the potential impact of CMV, we followed the procedure suggested by Mossholder et al. (1998) and compared a single-factor model with a multi-factor model. Model 1 yielded a chi-square value of 2726.328 (df = 252, *p* < 0.001), while Model 2 showed 424.834 (df = 224, *p* < 0.001). The results indicated that the single-factor model was not supported, suggesting that CMV was not a significant issue. Furthermore, according to Podsakoff et al. (2003), Harman’s single-factor test was conducted using principal component analysis. The unrotated results showed that the first factor accounted for 20.27% of the total variance, which is below the critical threshold of 50%.

### 3.5 Robustness and heterogeneity test

To examine whether the hypothesized paths are robust across key teacher characteristics, we conducted multiple regression analyses controlling for demographic and work-related variables, including gender, age, marital status, teaching experience, contract type, and weekly working hours. The dependent variable was stress self-management, and the main predictors were mindfulness, resilience, and cognitive reappraisal.

The regression model was significant, *F*(9, 277) = 19.526, *p* < 0.001, and explained 36.8% of the variance in stress self-management (adjusted R^2^ = 0.368). All control variables, gender, age, marital status, teaching experience, contract type, and weekly working hours, which were non-significant predictors (all *p* > 0.05), indicating that the effects of resilience and cognitive reappraisal on stress self-management were not confounded by these demographic or workload characteristics. Pearson correlation analyses among control variables and the main constructs further confirmed weak and mostly non-significant associations between covariates and the primary variables of interest (see Table 2).

**Table 2:** Pearson correlation analyses.

Variables	1	2	3	4	5	6	7	8	9	10
Gender	1									
Age	−0.148*	1								
Marital Status	−0.116*	0.725**	1							
Years of Teaching Experience	−0.118*	0.859**	0.647**	1						
Employment type	−0.001	−0.302**	−0.236**	−0.242**	1					
Average Weekly Working Hours	−0.130*	0.100	0.094	0.103	0.011	1				
Mindfulness	−0.108	0.045	0.024	0.078	0.092	−0.010	1			
Resilience	−0.200**	−0.013	−0.014	−0.011	0.068	−0.034	0.440**	1		
Cognitive reappraisal	−0.125*	−0.034	−0.017	0.015	0.108	−0.052	0.549**	0.672**	1	
Stress self-management	−0.133*	−0.001	−0.024	0.035	0.092	0.006	0.374**	0.556**	0.572**	1

**p < 0.01.

## 4 Result

### 4.1 Measurement

The measurement model exhibited a satisfactory overall fit (χ^2^/df = 1.897, NFI = 0.931, CFI = 0.966, TLI = 0.962, RMSEA = 0.056), meeting the recommended benchmarks (Hair et al., 2012). All standardized loadings were significant (*p* < 0.001) and ranged from 0.724 to 0.922, confirming that each item adequately represented its latent construct. As shown in Table 3, internal consistency was high across all constructs, with Cronbach’s alpha ranging from 0.895 to 0.948 and composite reliability (CR) between 0.897 and 0.949, exceeding the 0.70 threshold. The average variance extracted (AVE) values ranged from 0.593 to 0.787, all above the recommended minimum of 0.50, indicating satisfactory convergent validity. Mindfulness demonstrated the highest reliability (Cα = 0.948, CR = 0.949, AVE = 0.787), followed by resilience and cognitive reappraisal, while stress self-management also met the reliability and validity standards.

**Table 3:** Reliability and validity.

Items	Loadings	Cα	AVE	CR
Mindfulness		0.948	0.787	0.949
MIN1	0.844			
MIN2	0.890			
MIN3	0.901			
MIN4	0.922			
MIN5	0.877			
Resilience		0.943	0.738	0.944
RES1	0.775			
RES2	0.890			
RES3	0.880			
RES4	0.845			
RES5	0.869			
RES6	0.888			
Cognitive reappraisal		0.946	0.748	0.947
CR1	0.850			
CR2	0.795			
CR3	0.857			
CR4	0.880			
CR5	0.901			
CR6	0.902			
Stress self*-*management		0.895	0.593	0.897
SSM1	0.727			
SSM2	0.724			
SSM3	0.783			
SSM4	0.760			
SSM5	0.797			
SSM6	0.822			

Discriminant validity was further confirmed using the Fornell–Larcker criterion (Table 4). The square roots of AVE (diagonal elements) were greater than the corresponding inter-construct correlations (off-diagonal elements), demonstrating that all constructs were empirically distinct. Inter-construct correlations ranged from 0.374 to 0.672, suggesting moderate associations without redundancy. Additionally, skewness and kurtosis values for all variables were within ±2, indicating acceptable univariate normality. Collectively, these results provide strong evidence for the measurement model’s reliability, convergent validity, discriminant validity, and good overall fit, ensuring a sound foundation for subsequent structural analyses.

**Table 4:** Discriminant validity.

Construct	M	SD	|Skew|	|Kurtosis|	1	2	3	4
Mindfulness (1)	3.728	0.952	≤1.145	≤0.855	0.887			
Resilience (2)	3.470	0.850	≤0.474	≤0.621	0.440 **	0.859		
Cognitive Reappraisal (3)	3.753	0.814	≤1.240	≤1.829	0.549 **	0.672 **	0.865	
Stress Self-Management (4)	3.262	0.699	≤0.318	≤0.397	0.374 **	0.556 **	0.572 **	0.770

The square root of the AVE is in diagonals; off diagonals are Pearson’s correlations of constructs. **p < 0.01.

### 4.2 Structural model

**Figure 2:**
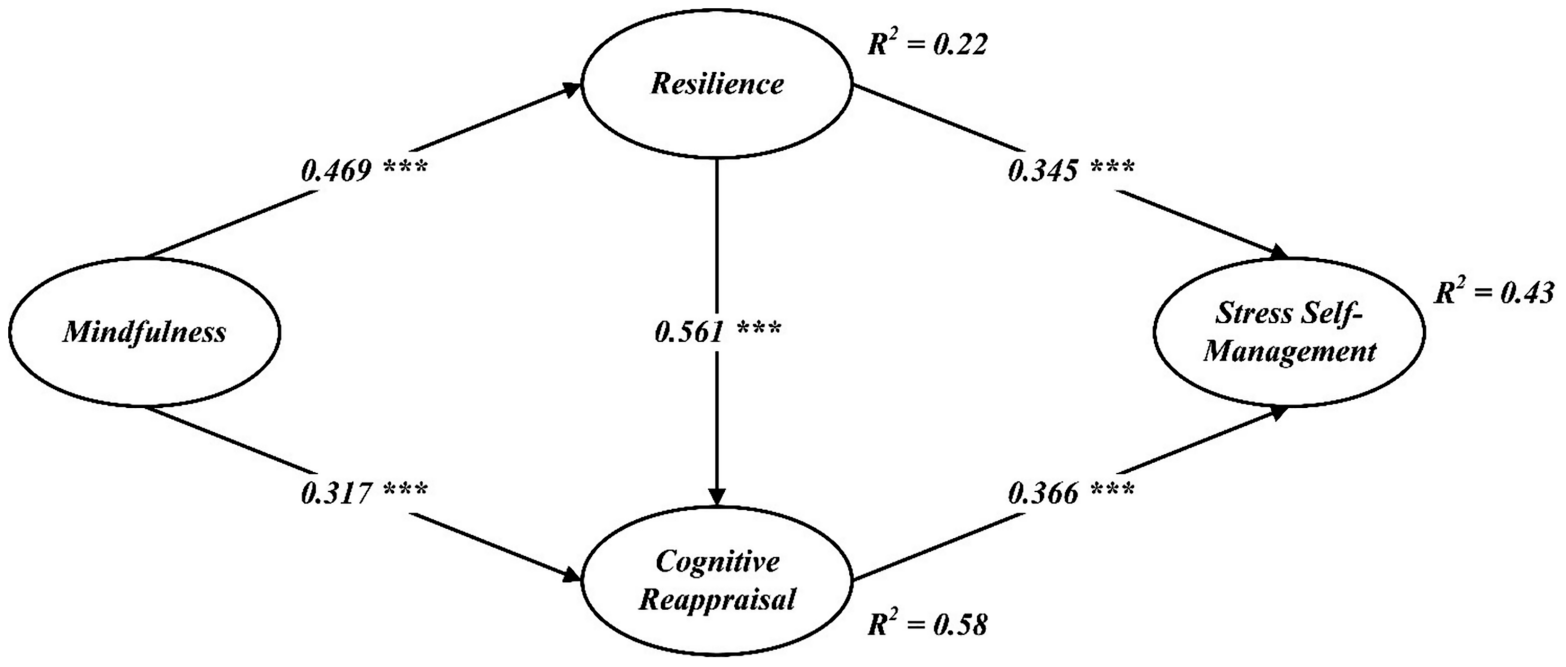
Structural model.

Based on the validated measurement model, the structural paths were estimated and all hypothesized associations were supported (Table 5; Figure 2). Specifically, mindfulness positively predicted resilience (*β* = 0.469, *p* < 0.001) and cognitive reappraisal (*β* = 0.317, *p* < 0.001), supporting H1–H2. Resilience showed significant positive effects on cognitive reappraisal (*β* = 0.561, *p* < 0.001) and stress self-management (*β* = 0.345, *p* < 0.001), supporting H3–H4. Cognitive reappraisal was positively associated with stress self-management (*β* = 0.366, *p* < 0.001), supporting H5. The model accounted for R^2^ = 0.22 (resilience), R^2^ = 0.58 (cognitive reappraisal), and R^2^ = 0.43 (stress self-management).

**Table 5:** Bootstrapping results.

Path	Point estimate	Product of coefficients	Bootstrapping		
			Bias-corrected 95% CI		Two-tailed significance
		SE	Lower	Upper	
Direct effect					
MIN → RES	0.469	0.064	0.338	0.586	*p* < 0.001
MIN → CR	0.317	0.060	0.202	0.436	*p* < 0.001
RES → CR	0.561	0.052	0.455	0.656	*p* < 0.001
RES → SSM	0.345	0.080	0.192	0.507	*p* < 0.001
CR → SSM	0.366	0.077	0.212	0.514	*p* < 0.001
Indirect effect					
MIN → RES	0.263	0.038	0.195	0.345	*p* < 0.001
MIN → SSM	0.374	0.053	0.270	0.475	*p* < 0.001
RES → SSM	0.205	0.047	0.122	0.309	*p* < 0.001
Total effect					
MIN → RES	0.469	0.064	0.338	0.586	*p* < 0.001
MIN → CR	0.580	0.064	0.445	0.695	*p* < 0.001
MIN → SSM	0.374	0.053	0.270	0.475	*p* < 0.001
RES → CR	0.561	0.052	0.455	0.656	*p* < 0.001
RES → SSM	0.550	0.055	0.438	0.653	*p* < 0.001
CR → SSM	0.366	0.077	0.212	0.514	*p* < 0.001

Indirect, sequential, and total effects were estimated using bias-corrected bootstrap (5,000 resamples, two-tailed). Table 5 reports 95% CIs, and *p*-values for the direct effect, the indirect effects, and the total effect. Results indicated a significant sequential mediation whereby resilience and cognitive reappraisal jointly transmitted the effect of mindfulness to stress self-management (standardized indirect effect = 0.374, *p* < 0.001; 95% CI not including zero), supporting H6.

To examine the robustness of the focal paths, we incorporated two key control variables, gender and age, into the model. The inclusion of these covariates did not materially alter the pattern or significance of the main effects. Model fit remained satisfactory (χ^2^/df = 1.846, NFI = 0.920, CFI = 0.962, TLI = 0.957, RMSEA = 0.041). All primary paths remained significant and in the expected directions: mindfulness → resilience (*β* = 0.382, *p* < 0.001), mindfulness → cognitive reappraisal (*β* = 0.270, *p* < 0.001), resilience → cognitive reappraisal (*β* = 0.586, *p* < 0.001), resilience → stress self-management (*β* = 0.261, *p* < 0.001), cognitive reappraisal → stress self-management (*β* = 0.269, *p* < 0.001). Both gender (*β* = −0.023, *p* = 0.676) and age (*β* = 0.011, *p* = 0.764) were non-significant predictors of stress self-management. These results indicate that the hypothesized relationships are robust and not confounded by these demographic characteristics.

## 5 Discussion

### 5.1 Theoretical contributions

Grounded in the COR theory, the present study systematically examined how mindfulness is associated with stress self-management among university faculty through the dual mediating roles of resilience and cognitive reappraisal. The findings extend theoretical understanding of psychological adaptation processes in higher education settings and offer several contributions to the existing literature.

First, this study extends the application of COR theory (Hobfoll et al., 2018) to the context of higher education by empirically identifying a multi-level psychological mechanism involving mindfulness, resilience, and cognitive reappraisal. While previous COR-based studies have primarily focused on healthcare workers, first responders, or corporate employees (Halbesleben et al., 2014), empirical research involving university faculty has been scarce. By illustrating how teachers mobilize and transform internal psychological resources when facing professional stressors, this study enriches the explanatory scope of COR theory within academic environments characterized by chronic and multidimensional pressures.

Second, the results provide novel insights into mindfulness as a foundational psychological resource that supports the acquisition and deployment of secondary resources such as resilience and cognitive reappraisal. Although prior literature has documented the link between mindfulness and well-being (Minh Anh Quang et al., 2022), relatively few studies have explored how mindfulness contributes to resource gain spirals as conceptualized by COR theory (Hobfoll et al., 2018). The present findings suggest that mindfulness is positively associated with both resilience and cognitive reappraisal, thereby highlighting its central role in initiating and sustaining adaptive resource development rather than implying a unidirectional causal effect.

Third, by simultaneously examining resilience and cognitive reappraisal as dual mediators, this study addresses an existing theoretical gap. Previous studies have often examined these constructs in isolation, without integrating their sequential or interactive dynamics in stress adjustment processes (Jiang et al., 2022). The identification of a dual mediation pathway provides a more integrative and process-oriented understanding of how psychological resources may operate synergistically to shape stress-related outcomes. It is important to note that emotion regulation in this study was operationalized specifically through cognitive reappraisal; other regulation strategies such as suppression were not assessed. Thus, the results speak to reappraisal-based regulatory processes within the COR framework rather than the full spectrum of emotion regulation.

Moreover, these findings complement recent China-based evidence emphasizing alternative psychological routes from mindfulness to well-being. For instance, Wu et al. (2025) identified dual pathways through self-compassion and reduced rumination among Chinese university students, reflecting affective and cognitive mechanisms. The current study, focusing on resilience and reappraisal among university faculty, highlights a distinct but complementary regulatory chain that may characterize educators’ adaptive responses to occupational stress. Together, these strands of evidence suggest that mindfulness can foster well-being in Chinese educational settings through multiple, potentially trainable mechanisms spanning affective, cognitive, and regulatory domains.

Finally, these findings align with and extend recent China-based evidence emphasizing diverse psychological routes from mindfulness to well-being. Recent studies among Chinese populations have identified multiple mediating mechanisms such as self-compassion, reduced rumination, and resilience that jointly explain how mindfulness promotes emotional and occupational well-being (Wu et al., 2025; Li et al., 2023; Pang et al., 2025; Wang et al., 2023). Collectively, these studies suggest that mindfulness exerts its benefits primarily through affective and cognitive regulatory processes rather than through direct effects. Building on this growing body of work, the present study focuses on resilience and cognitive reappraisal among university faculty, illustrating a distinct yet complementary regulatory chain that characterizes educators’ adaptive responses to occupational stress. This extension enriches the understanding of mindfulness-based mechanisms within Chinese educational contexts and suggests that mindfulness may foster well-being through multiple, trainable psychological resources.

In sum, this study enhances understanding of the interrelations among mindfulness, resilience, and cognitive reappraisal, extends the reach of COR theory to the underexplored population of university faculty, and highlights a culturally relevant, resource-based model of psychological adaptation in the Chinese higher education context.

### 5.2 Practical implications

The findings of this study provide meaningful implications for enhancing the psychological well-being, adaptive coping, and overall performance of university faculty, as well as for informing institutional strategies for mental health promotion in higher education.

First, establish mindfulness as a foundational resource. Mindfulness demonstrated strong positive associations with both resilience and cognitive reappraisal, indicating its central role in fostering adaptive psychological capacities. Universities can integrate mindfulness into routine faculty development through multiple channels, such as guided meditation sessions during departmental meetings, short mindfulness exercises delivered via mobile apps, or workshops embedded in teacher training curricula. Beyond formal sessions, micro-practices like mindful breathing before lectures, brief reflective pauses between meetings, or journaling exercises can be promoted to encourage daily engagement. Embedding mindfulness into institutional routines helps normalize mental health practices, reduces stigma, and signals organizational support. Leadership endorsement is crucial to foster participation and signal that mindfulness is a valued professional skill. Institutions might also offer incentives, such as recognition of participation in professional development records, to enhance motivation and sustained engagement.

Second, build resilience skills as a progressive extension of mindfulness training. The sequential model suggests that resilience bridges mindfulness and cognitive reappraisal, strengthening adaptive stress management. Universities can implement targeted resilience-building programs that include reflective exercises to process challenging teaching experiences, scenario-based workshops simulating academic or administrative stressors, peer mentoring groups to share coping strategies, and collaborative problem-solving sessions to build social support networks. These activities not only enhance individual coping capacities but also foster a supportive and resourceful faculty culture. Emphasizing continuous skill development rather than one-off stress-relief sessions ensures faculty internalize adaptive strategies and gradually strengthen their ability to manage professional pressures. Institutions could further encourage faculty to set personal resilience goals, track progress over time, and participate in inter-departmental resilience challenges to create an engaging, community-based approach.

Third, enhance cognitive reappraisal routines through modular, sequential training. Consistent with the quasi-experimental MBSEL curriculum reported by Wu et al. (2025), cognitive reappraisal can be developed through structured modules that build on prior mindfulness and resilience training. Programs can be implemented in a stepwise fashion: starting with foundational mindfulness sessions to cultivate awareness and reduce automatic emotional reactivity, followed by resilience workshops to strengthen coping and problem-solving abilities, and finally cognitive reappraisal training to refine emotion regulation strategies. Practical exercises could include guided cognitive restructuring of academic setbacks, scenario-based role plays simulating student or colleague interactions, reflective journaling of emotionally challenging events, and peer discussions to explore alternative interpretations of stressful situations. Integrating these practices into pre-service teacher education and ongoing in-service programs enables faculty to consolidate skills progressively, reinforcing the accumulation of adaptive psychological resources. Such modular and sequential design ensures that each skill builds upon the previous one, promoting sustained improvements in stress self-management and overall well-being.

In sum, these implications highlight a strategic shift from reactive, short-term stress mitigation to proactive, evidence-informed psychological resource development. By establishing mindfulness as a foundation, progressively enhancing resilience, and refining cognitive reappraisal through structured modules, universities can position faculty well-being as an integral component of academic governance, ultimately fostering a more resilient, adaptive, and high-performing educational workforce.

### 5.3 Limitations

Despite its contributions, this study is subject to several limitations that should be addressed in future research. First, the use of cross-sectional data limits causal inference regarding the relationships among mindfulness, resilience, cognitive reappraisal, and stress self-management; short multi-wave, longitudinal, or experimental designs (e.g., experience sampling) would better establish temporal order and more rigorously test the proposed sequential pathways. Second, all variables were assessed through self-reports, which may introduce social desirability and common-method variance; moreover, our operationalization captured dispositional mindfulness rather than skills-based, instructor-guided practice and should not be conflated with “positive thinking.” Future work should adopt multi-method approaches (behavioral tasks, physiological indicators, peer/supervisor ratings) and incorporate training fidelity, practice dosage/adherence, and active comparators (e.g., stress-management education) to isolate mindfulness-specific effects. Third, the sample comprised university faculty from a single Chinese province, which may limit generalizability; replication across regions, institution types, and cultures is needed to examine boundary conditions. Finally, the model tested only two mediators—resilience and cognitive reappraisal; future studies could include additional mechanisms (e.g., self-efficacy, decentering, emotion-regulation flexibility) and contextual resources (e.g., perceived organizational support). We also acknowledge that the present contribution is incremental in content and perspective; future research should pursue stronger novelty via preregistered longitudinal/experimental designs and objective practice data to unpack dose–response relations. Taken together, clarifying construct–practice distinctions, strengthening causal identification, broadening sampling frames, and expanding mechanisms and boundary conditions will enhance both the conceptual precision and practical relevance of this line of work.

## Data Availability

The raw data supporting the conclusions of this article will be made available by the authors, without undue reservation.
